# Inhaled corticosteroids for abnormal pulmonary function in children with a history of Chronic Lung Disease of Infancy: study protocol [ISRCTN55153521]

**DOI:** 10.1186/1471-2466-5-6

**Published:** 2005-04-12

**Authors:** Saad Alotaibi, David Johnson, Mark Montegomery, Reginald Sauve, Sheldon Spier

**Affiliations:** 1 Pediatric Pulmonologist, Pediatric Department, Farwanyah Hospital, Kuwait; 2 Pediatric Emergency Consultant, Pediatric Emergency Department, Alberta Children Hospital, University of Calgary, Calgary, Canada; 3 Pediatric Pulmonology Consultant, Pediatric Pulmonology Division, Alberta Children Hospital, University of Calgary, Calgary, Canada; 4 Community Health Sciences, Faculty of MEDICINE (Medical School) Graduate Science Education, Paediatrics, University of Calgary, Calgary, Canada; 5 Head of Pediatric Pulmonology Division, University of Calgary, Alberta Children Hospital, Calgary, Canada

## Abstract

**Background:**

There is considerable evidence from the literature that children with chronic lung disease of infancy (CLD) have abnormal pulmonary function in childhood and this could have an impact on their life quality and overall health. There are similarities between CLD and asthma, and corticosteroids are the mainstay treatment for asthma. Many physicians use inhaled corticosteroids in children with CLD with no evidence. Therefore we wish to conduct a randomized double-blinded placebo controlled trial to test for the role of inhaled corticosteroids in children aged from3 to 9 years with a history of CLD. Our primary hypothesis will be that inhaled corticosteroids are beneficial in children with CLD.

**Methods:**

Our primary hypothesis is that using inhaled steroids; Beclomethasone Dipropionate (QVAR) 100 mcg 2 puffs 2 times a day for 6 weeks will improve the respiratory system resistance and the quality of life in children with CLD.

**Discussion:**

We propose that Beclomethasone Dipropionate (QVAR) will affect the pulmonary function after 6 weeks of treatment. In summary we think that our study will highlight knowledge on whether the use of inhaled steroids is clinically effective for CLD.

## Background

Children with a history of severe CLD were left with significant pulmonary sequelae at school age compared with prematurely born children who had required ventilator assistance and supplemental oxygen in the neonatal period but who did not go on to have CLD [1]. It was also documented that prematurely born children with respiratory distress syndrome (RDS) have long term pulmonary sequelae consisting of airway obstruction and airway hyper reactivity, which are related to both the severity of the initial pulmonary insult and its treatment as well as to a familial predisposition to airway hyper reactivity. The role of inhaled corticosteroids in children with chronic lung disease of infancy or bronchopulmonary dysplasia was not previously studied although most if not all physicians use this treatment for these children whenever they develop chest infections or wheezy episodes. This is a randomized double blinded placebo controlled study using inhaled corticosteroid (CFC free Beclomethasone dipropionate, QVAR) for 6 weeks and measuring the effect on pulmonary function and quality of life.

## Methods

We will use a double blind randomized parallel design. Patients will be invited to the study by sending a letter from the perinatal clinic office inviting them to contact us. Consent will be handed to the parents at the beginning of the study. Subject randomization will be carried out using a computer generated random numbers. Medications and placebo will be kept locked in a cabinet in the clinic area, one of the clinic nurses will be responsible for dispensing the medication or placebo. The medication or placebo will be taken twice daily, at 08:00 and 20:00. The Placebo will have the same taste, shape and color of the study drug. There will be a run in period for 1 week before the study, for teaching the patients, and checking proper technique, and filling out the Child Health Questionnaire (CHQ). In the first visit, informed consent filled, inclusion and exclusion criteria will be obtained. Medical and surgical history, physical exam, inhalation and pulmonary function technique will be done. We will review the daily diary, and prepare the placebo for all patients in the run in period. One week after the run in period, randomization carried out and patients entered into the study. Phone calls will be carried out every two weeks to optimize compliance. After six weeks of treatment, CHQ will be filled. Forced expiratory volume at 1 second (FEV1) (for children older than 6 years of age) and respiratory system resistance at 5 Hz frequencies will be measured.

The Impulse Oscillometry tool to be used is developed by Jaeger Company (Wurzburg, Germany). In short rectangular mechanical impulses containing the whole frequency spectrum are applied to the respiratory system by a mouthpiece, while the patient is breathing quietly. The resulting pressure and volume signals are analysed for their amplitude and phase difference to determine the Rrs at 5 Hz of the total respiratory system [2]. One investigator will perform all pulmonary function tests. After a short explanation, the children will perform some practice trials. When the child is feeling comfortable, a measurement of 25–35 seconds will be started. Patients will be asked to breathe quietly through a round mouthpiece. A nose clip will be used and the cheeks will be supported by the hands of the investigator to minimize pressure loss through the upper airway shunt. Measurement will be discarded if the time-flow pattern showed apnea, speaking, swallowing or air leak, as described by Bisgraaed and Klug. The following parameter Rrs at 5 Hz will be recorded before and 20 minutes after administration of 200 mcg salbutamol. The spacer device used to administer the medication will be an aerochamber with mask for children under the age of 4.7 yrs old and with a mouth piece for children above the age of 4.7 yrs [3-5].

The Study of a patient should be delayed six weeks if developed a viral respiratory illness. Bronchodilators should not be used on the day of the study visit (at least 24 hours for long acting, 6 hours for short acting). Neonatal variables to be collected from medical records: gender, birth weight, birth length, head circumference, gestational age, neonatal treatment, total days on oxygen, maximal FiO2, days on CPAP, days on IPPV, use of steroids (how much and how long), and the use of surfactant. Maternal data: parity, living offspring's, and smoking days during pregnancy. Skin prick testing for the 10 common allergens will be done at the start of the study. Atopy will be defined by one or more positive reactions (3 mm wheal) to 10 common aeroallergens. Parents will fill out daily symptom scores. Social and environmental data to be collected are smoking habits, pets, family history of asthma and asthma in the child. Methacholine challenge testing will be done in the start of the study to define those patients with airway hyper reactivity, which might be a sensitive indicator of response to steroids [6,7]. Respiratory function will be measured using Spirometry (FEV1) and Impulse Oscillometry (RS @ 5 Hz). Quality of life will be measured using the CHQ. Treatment group will receive QVAR 100 mcg 2 puffs twice daily; placebo will be given to the other arm for 6 weeks, after which the respiratory function and quality of life will be measured.

### Aim of the study

To study the effect of inhaled steroids on school- aged children with CLD.

### Primary hypothesis

Inhaled corticosteroids, Beclomethasone Dipropionate will improve respiratory system resistance after 6 weeks of treatment.

### Primary outcome measures

Improvement in respiratory system resistance at 5 Hz frequency measured by impulse oscillometry after 6 weeks of inhaled corticosteroids in CLD.

### Secondary outcome measures

Improvement in Quality of life system score using Child Health Questionnaire (CHQ) and FEV1 response to bronchodilators (Salbutamol) after inhaled corticosteroids treatment for 6 weeks in children with CLD.

The inclusion criteria: children between 3 and 9 years of age with a history of CLD with the following criteria.

1. Premature birth at 36 weeks gestation or less.

2. Clinical and radiological diagnosis of CLD. Patient charts will be reviewed and the x-rays will be reviewed, even if there is no x-ray findings patients will be included if they fulfil the other criteria.

3. Requirement of supplemental oxygen to maintain saturation of 90%, for at least 36 wk corrected gestation.

The exclusion criteria:

1. Cardiovascular disease other than PDA (Patent Ductus Arteriosus)

2. Those with severe neurological disease, developmental delay. Oxygen requirement for respiratory diagnosis other than CLD e.g. congenital diaphragmatic hernia and aspiration. An inability to perform spirometry or IOS, or noncompliance with therapy and those with pneumonia.

### Statistical consideration

The required number of patients was estimated as n = 74 (37 in each group) and to allow 10% dropout we need 82 patients. The sample size estimation was based on a type I error rate of = .05 and a type II error rate of = 0.20. The improvement in airway resistance at 5 HZ as primary end point was expected to be 15% for the treatment group. The coefficient variation for the impulse oscillometry measurement is 10%. The primary statistical analysis of efficacy data is based on the intention-to-treat patients, who were randomly assigned to treatment and received at least one dose of medication. Baseline characteristics will be compared using descriptive statistics including confidence intervals. Imbalance between the groups on key variables, which might influence the observed outcome, will indicate that an adjusted analysis is necessary using these variables. The differences between airway resistance at 5 HZ response will be tested with a 2-tailed paired test on a significance level of = .05. Analysis of FEV1 improvement will be performed by two-tailed T test (= .05). All tests, except those for CHQ, will be done by SSPS procedures. For CHQ analysis, the differences of sum scores at week 6 after treatment to baseline will be tested by Wilcoxon rank sum test using StatXa.

## Discussion

Bronchopulmonary Dysplasia (BPD) is a chronic lung disease related to injury induced by mechanical ventilation and oxygen therapy in prematurely born infants. William Northway, a radiologist at Stanford University, described this pathology in 1967[8]. More recently the term chronic lung disease (CLD) has been used since nowadays preterm infants with birth weights between 500 and 750 grams have at least a 50 percent chance of survival at 24 weeks gestation age[9]. The clinical diagnosis of this CLD is made at one month of age in prematurely born infants who have undergone mechanical ventilation for at least one week, who have symptoms of persistent respiratory distress, are dependent on supplemental oxygen, and who have chest radiographs that show rounded radio lucent areas in the lung alternating with thinner strands of radiodensity.

The pathogenesis of CLD is believed to be multifactorial, with pulmonary oxygen toxicity and barotrauma having important roles [10]. More recently inflammatory mediators, cytokines, and proteolytic enzymes released by inflammatory cells, whose influx into the lungs is the consequence of oxygen exposure and prenatal or postnatal sepsis, were described [11]. The release of neuropeptides such as substance P from afferent nerve endings in the airways or lung parenchyma may accelerate this inflammatory cascade[12]. Coates et al found decreased expiratory flows in children 7 and 8 years old who had survived the respiratory distress syndrome, and later suggested that oxygen therapy could have been at least partly responsible for these sequelae [13]. CLD has become the most common form of chronic lung disease in infants in the United States with approximately 7000 new cases occurring each year [14].

It has been noted that infants with BPD were twice as likely to be hospitalized in the first 2 years of life compared with preterm infants without BPD [15]. But at age 7 years both groups had more cough and wheeze than the term group. Pulmonary function including lung volumes and expiratory airflow were worse in the BPD group. This group showed more evidence of gas trapping and hyperinflation and significant expiratory airflow obstruction [15]. This was confirmed by the study done by Jacob et al who compared children of BPD at 11 years old with children in a control group who were born preterm but who did not have BPD [9]. This was also supported by the findings of Coates et al who confirmed that premature birth and the development of the RDS of the newborn have been associated with long term pulmonary sequelae [13,16]. Is there any treatment available for those children?

The literature suggested that there is significant morbidity and mortality from chronic lung disease in infancy into adult and elderly ages. In a follow up study of men born during 1911–1930 whose birth weight, weight at 1 year, and childhood respiratory illnesses were recorded at that time by health visitors [17], Barker et al found that lower birth weight was associated with worse adult lung function. Bronchitis, pneumonia, or whooping cough in infancy further reduced adult lung function, they concluded that death rates from chronic obstructive disease, but not from another disease related to smoking or lung cancer fell with increasing birth weight and weight at 1 year. This was supported by the findings of Peto et al [18] in their follow up study of men whose lung function and respiratory symptoms had been assessed in adult life and showed that the risk of death from chronic obstructive airway disease was strongly correlated with the initial FEV1.

Liggins and Howie have demonstrated that the administration of a corticosteroid leads to a decreased incidence of RDS in human premature infants[19]. It is well known that inhaled and systemic steroids are used for the prevention of CLD in infancy [20]. Any pulmonary growth retardation would be expected to have an effect on the airways and result in increased airway resistance and lower flows. It was pointed out that the only major changes in airways from birth to adulthood are in their size [21]. High incidence of asthma has been noted in families of neonates in whom the RDS was complicated by CLD [22]. Other clinical studies suggest an increased incidence of airway hyper reactivity in prematurely born children [23-26]. Recurrent wheeze in childhood and readmission to hospital for bronchiolitis like illness has been observed among prematurely born children who survived the RDS [24]. Most patients with CLD also have increased airway lability and improvement in expiratory flow rates following bronchodilator inhalation suggesting that such therapy may improve pulmonary function in these children [15]. It was anticipated that the incidence of CLD would decrease after the introduction of surfactant therapy, clinical trials by Horbar and Long failed to confirm this [27,28].

So far there are no studies have been published studying the clinical response to inhaled corticosteroids in children CLD. Pulmonary function testing for these children can be done using different techniques. Pennings et al [29] used forced oscillation technique (FOT) to detect changes in airway bronchial tone of asthmatic patients, that were not detected by conventional flow volume recording. It was found that the changes of the diameter of small airways of children could be missed by routine spirometry. The apparent increase incidence of bronchiolitis in RDS survivor's points to these small airways as a source of pathology [30]. In 1956, Dubios et al described the use of FOT as a measure of total respiratory system impedance, which was non invasive and required minimal cooperation from the subject and it can be applied to infants and older children. Laberge et al used FOT to measure respiratory system resistance in 3 to 6 years old asthmatic children and they concluded that the dry powder inhaler (Turbuhaler) and the metered dose inhaler with spacer (Nebuhaler) are equally effective in administering terbutaline [31].

## Conclusion

We propose that Beclomethasone Dipropionate (QVAR) will affect the pulmonary function after 6 weeks of treatment. In summary we think that our study will highlight knowledge on whether the use of inhaled steroids is clinically effective for CLD. This study was approved by the conjoint Health Research Ethics Board and Child Health Research Committee at the University of Calgary, Alberta, Canada.

## List of abbreviations

CLD = chronic lung disease of infancy.

CHQ = child health questionnaire.

FEV1 = forced expiratory volume at 1 second.

Rsr = respiratory system resistance.

IOS = impulse oscillometry.

## Competing interests

The authors declare that they have no competing interests. This study had a gift of fund from 3 M pharmaceuticals-Canada in the form of a payment of CAD$ 6,400.00 as well as 100 canisters of 3M QVAR™ 100 ug and 100 canisters of placebo.

## Authors' contributions

The first author as research pulmonology fellow collected the literature and writing, editing the manuscript and as organiser of this study and patient recruitment. Second author gave statistical help of this study protocol. Third author helped in the design of the study. Forth author gave ethical help for the study and recruitment of patients form perinatal clinic. Fifth author was the principal investigator in this clinical fellow research study protocol, also contributed to the editing and writing of the manuscript. All authors contributed to the study protocol from all aspects.

## Pre-publication history

The pre-publication history for this paper can be accessed here:


